# *Lacticaseibacillus rhamnosus* WH.FH-19: Probiotic Traits, Fermentation Performance, and Potential for Functional Fermented Milk Production

**DOI:** 10.3390/foods15020271

**Published:** 2026-01-12

**Authors:** Shiyuan Sun, Lu Feng, Liping Sun, Xuemei Zhu, Mo Zhou, Xinling Li, Guangqing Mu

**Affiliations:** 1 School of Food Science and Technology, Dalian Polytechnic University, Dalian 116034, China; 2 College of Food Science and Engineering, Tarim University, Alaer 843300, China; 3 Production & Construction Group Key Laboratory of Special Agricultural Products Further Processing in Southern Xinjiang, Alaer 843300, China; 4 Urumqi Dairy Industry Association, Urumqi 830000, China

**Keywords:** *Lacticaseibacillus rhamnosus*, probiotic, safety assessment, synergistic fermentation, sensory quality

## Abstract

*Lacticaseibacillus rhamnosus* WH.FH-19 exhibits robust probiotic and technological traits for fermented dairy applications. *L. rhamnosus* WH.FH-19 shows superior functional potential compared to the benchmark strain *Lacticaseibacillus rhamnosus* GG. Kinetic studies confirm *L. rhamnosus* WH.FH-19’s vigorous growth and rapid acidification kinetics in bovine milk. In vitro characterization reveals enhanced probiotic properties, including significantly greater epithelial adhesion, tolerance to gastrointestinal stresses, cholesterol assimilation capacity, and antioxidant activity. Comprehensive safety assessment demonstrated the absence of hemolysis, sensitivity to clinically relevant antibiotics, and negligible tyramine production. Optimal synergistic fermentation with *L. bulgaricus* CICC 6047 and *S. thermophilus* CICC 6038 was achieved using a defined inoculum ratio. Under these conditions, *L. rhamnosus* WH.FH-19 specifically potentiated the activity of the *S. thermophilus* strain, accelerating fermentation kinetics without subsequent post-acidification while improving product sensory attributes. These findings establish *L. rhamnosus* WH.FH-19 as a safe, functionally robust probiotic with significant technological benefit for commercial fermented dairy production.

## 1. Introduction

The increasing global demand for functional dairy foods has accelerated the search for probiotic strains that combine robust fermentation performance with verified health-promoting properties. Lactic acid bacteria (LAB), including *Streptococcus*, *Lactococcus*, *Lactobacillus*, *Pediococcus*, and *Leuconostoc* [1], have long served as traditional dairy starters. However, *Lacticaseibacillus rhamnosus*, a species widely recognized for its probiotic potential [2], remains underutilized in commercial dairy fermentations due to its weak lactose utilization and poor proteolytic activity. Beyond their metabolic and ecological roles in the gut, probiotic LAB are increasingly expected to deliver strain-specific health benefits such as antioxidant activity, cholesterol reduction, and immune regulation, making their integration into dairy platforms a compelling goal for the functional food industry.

The most extensively studied strain, *L. rhamnosus* GG, has demonstrated clinical benefits such as immune enhancement [2], cholesterol reduction [3], and gastrointestinal survival and transient ileal/colonic colonization [4]. However, *L. rhamnosus* GG exhibits limited growth and acidification in milk matrices, which impairs its independent use as a starter culture [5]. This metabolic limitation restricts its application in large-scale dairy processing, motivating efforts to identify new *Lacticaseibacillus rhamnosus* strains with dual functionalities of technological viability and probiotic efficacy. To address this knowledge gap, a strain of *Lacticaseibacillus rhamnosus*, designated WH.FH-19 was isolated from traditionally fermented camel milk in Xinjiang, China. This strain exhibits robust dairy fermentation capabilities and enhanced probiotic attributes. In previous studies [6], it has been demonstrated that *L. rhamnosus* WH.FH-19 can increase the content of flavor substances, promote the metabolism of amino acids and carbohydrates in fermented milk, and produce more antioxidant metabolites, which have the potential to be a starter culture for fermented milk. Unlike traditional starter cultures that focus solely on fermentation performance, *L. rhamnosus* WH.FH-19 was designed to meet dual functional and technological criteria, enabling simultaneous health benefit delivery and process efficiency.

This study aimed to comprehensively evaluate its growth kinetics, acidification behavior, gastrointestinal tolerance, cholesterol assimilation, antioxidant capacity, and biosafety profile. Furthermore, its synergistic effects in co-fermentation with commercial starters (*S. thermophilus* and *L. bulgaricus*) were investigated, with a focus on fermentation performance, post-acidification behavior, and sensory attributes. Through this work, we propose *L. rhamnosus* WH.FH-19 as a next-generation multifunctional probiotic starter with both scientific and industrial significance.

## 2. Materials and Methods

### 2.1. Bacterial Strains and Fermentation Preparation

*Lacticaseibacillus delbrueckii* subsp. *bulgaricus* CICC 6047 (LB) and *Streptococcus thermophilus* CICC 6038 (ST) were from the China Center of Industrial Culture Collection (Beijing, China). *Lacticaseibacillus rhamnosus* WH.FH-19 (LR) was provided by Xinjiang Wangyuan Camel Milk Industrial Co., Ltd. (Xinjiang, China), derived from traditional fermented dairy products. It was the same strain as the one in Feng’s study [6]. *Lacticaseibacillus rhamnosus* (LGG) was preserved in the Dalian Key Laboratory of Functional Probiotic and Protein (Dalian, China).

All strains were routinely cultivated in MRS (Man Rogosa Sharpe) broth (Solarbio, Beijing, China) at 37 °C for 24 h under aerobic conditions. Bacterial cells were harvested by centrifugation (8000× *g*, 5 min, 4 °C), washed twice with phosphate-buffered saline (PBS, 0.01 mol L^−1^, pH 7.2), and resuspended in PBS to a concentration of approximately 1 × 10^8^ CFU mL^−1^ for inoculation.

Skim milk powder (Anchor, Fonterra Ltd., Auckland, New Zealand) was reconstituted to 12% (*w*/*v*) in distilled water and supplemented with 5% (*w*/*v*) sucrose. Adding sucrose to the skimmed milk powder basal medium serves the main purpose of providing an additional and readily available carbon source, which facilitates the rapid utilization of the carbon source and optimizes the growth, acid production, or synthesis of specific metabolites (such as extracellular polysaccharides) of lactic acid bacteria [7]. The mixture was subjected to heating in a boiling water bath at 100 °C for 30 min, cooled to fermentation temperature (40 °C), and inoculated with different starter combinations as required for monoculture or co-culture fermentation experiments. Unless otherwise stated, monoculture fermentation used *L. rhamnosus* WH.FH-19 or LGG at 1 × 10^8^ CFU mL^−1^. Fermentation was conducted at 40 °C until curd formation, after which samples were stored at 4 °C for downstream analysis.

### 2.2. Growth Kinetics and Fermentation Performance

The growth dynamics of *L. rhamnosus* WH. FH-19 and reference strain LGG in milk were evaluated by monitoring viable cell counts and titratable acidity over the fermentation period. Viable bacterial counts were determined by serial dilution and pour-plating on MRS agar, followed by incubation at 37 °C for 48 h. Results are expressed as colony-forming units per milliliter. The same enumeration method was also applied to samples taken at regular intervals during the subsequent 21-day refrigerated storage period to assess shelf-life stability.

Titratable acidity was determined by potentiometric titration (T5 automatic potentiometric titrator, Mettler-Toledo, Greifensee, Switzerland). Fermented milk samples were homogenized, and 10 g aliquots were mixed with 20 mL of distilled water. The mixture was titrated with a pre-calibrated 0.1 mol L^−1^ NaOH solution to a pH endpoint of 8.3 [8].

### 2.3. In Vitro Probiotic Properties

#### 2.3.1. Determination of the Autoaggregation Ability of the Strain

The method was modified slightly based on the previous one [9]. Selected strains were cultured in MRS broth at 37 °C for 18 h in a constant temperature incubator (Model DNP-9082, Shanghai Jinghong Laboratory Equipment Co., Ltd., Shanghai, China). Cells were then harvested by centrifugation (Model 5424R, Eppendorf AG, Hamburg, Germany) at 5000× *g* for 5 min, and washed twice with phosphate-buffered saline (PBS). Bacterial pellets were resuspended in PBS to 10^8^ CFU mL^−1^. For measurement, 4 mL aliquots of the suspension were vortex-mixed for 10 s using a vortex mixer (Model VORTEX-5, Haimen Qilinbeier Instrument Manufacturing Co., Ltd., Haimen, China), and the initial absorbance (A_1_) was immediately measured at 600 nm using a multifunction microplate reader (Thermo Scientific, Thermo Fisher Scientific, Vantaa, Finland). Following static incubation at 37 °C for 5 h, supernatant absorbance (A_2_) was measured. The calculation formula of auto-aggregation was as follows:

$$Autoaggregation\left(\%\right)=\left(1-\frac{A_{2}}{A_{1}}\right)\times 100$$

where A_1_ is the absorbance value of the cells at 0 h, and A_2_ is the absorbance value of the cells at 5 h.

#### 2.3.2. Determination of Strain Hydrophobicity

Bacterial suspensions (10^8^ CFU mL^−1^) were prepared as per Section 2.1. Hydrophobicity was assayed following Qureshi et al. [10], with modifications: 2 mL bacterial suspension was combined with 2 mL xylene, vortexed for 5 min, and incubated statically (37 °C, 30 min). The absorbance of the aqueous phase was measured at 600 nm. The calculation formula of hydrophobicity is as follows:

$$Hydrophobicity\left(\%\right)=\left(1-\frac{A_{4}}{A_{3}}\right)\times 100$$

where A_3_ is the absorbance of lactic acid bacteria before xylene treatment, and A_4_ is the absorbance of lactic acid bacteria after xylene treatment.

#### 2.3.3. Determination of the Adhesion Ability of the Bacterial Strain to HT-29 Cells

Bacterial adhesion to HT-29 cells was assessed following Tarrah et al. [11]. Adherent bacteria in 20 random microscopic fields were enumerated. Strains were categorized as follows: non-adhesive (<40 total bacteria), adhesive (41–100 bacteria), and strongly adhesive (>100 bacteria). Triplicate experiments with triplicate technical replicates were performed; data represent mean ± SD.

#### 2.3.4. Determination of the Survival Ability of the Strain in Simulated Gastric Fluid

Gastric and intestinal tolerance was assessed following Zhang et al. [12], with modifications. Activated cultures were harvested at 25 °C (3500× *g*, 5 min), washed twice with PBS, and resuspended in simulated gastric fluid (SGF: 0.3% *w/v* pepsin in PBS, pH 3.0). After 3 h incubation at 37 °C, viability was assessed by plate count. Cells were then pelleted (3500× *g*, 5 min), resuspended in simulated intestinal fluid (SIF: 0.3% *w/v* bile salts + 0.3% *w/v* trypsin in PBS, pH 8.0), and incubated for 8 h at 37 °C before final viability enumeration. The calculation formula of livability is as follows:

$$Livability\left(\%\right)=\frac{\log N_{1}}{\log N_{0}}\times 100$$

where N_1_ is the viable bacterial count (CFU mL^−1^) of simulated artificial gastric and intestinal fluids, and N_0_ is the initial viable bacterial count (CFU mL^−1^).

#### 2.3.5. Determination of Cholesterol-Lowering Capacity of Strains

Cholesterol assimilation capacity was assessed as follows: Solid cholesterol and beef bile salts were dissolved in anhydrous ethanol to yield a 15 mg mL^−1^ cholesterol ethanol solution (4.5% *w*/*v* bile salts). This solution was combined with MRS broth (1:15 *v*/*v*) to prepare cholesterol MRS medium (final concentrations: 0.1% *w/v* cholesterol, 0.3% *w*/*v* bile salts). Activated cultures were inoculated (2% *v*/*v*) and incubated at 37 °C for 24 h. After centrifugation at 4 °C (12,000× *g*, 10 min), supernatants were collected, and cholesterol was quantified using a commercial assay kit. Total cholesterol levels were measured using a Total Cholesterol Assay Kit (catalog no. A111-1-1) from Nanjing Jiancheng Bioengineering Institute (Nanjing, China). The calculation formula of the cholesterol degradation rate is as follows:

$$Cholesterol\;degradation\;rate\left(\%\right)=\frac{m_{0}-m_{1}}{m_{0}}\times 100$$

where m_0_ is the cholesterol content in the medium before fermentation, and m_1_ is the cholesterol content in the medium after fermentation.

#### 2.3.6. Determination of Antioxidant Capacity of Strains

Antioxidant capacity was assessed following Ragul et al. [13], with modifications. DPPH radical scavenging capacity: Samples were mixed 1:1 (*v*/*v*) with 0.22 mM DPPH in anhydrous ethanol, vortexed, and incubated at 25 °C in the dark for 30 min. Following centrifugation at 25 °C (4000× *g*, 10 min), supernatant absorbance was assayed at 517 nm. Absorbance was measured using a multifunction microplate reader (Thermo Scientific, Thermo Fisher Scientific, Finland) equipped with a quartz cuvette module.

The ABTS working solution was prepared by mixing 2 mM ABTS and 2.45 mM potassium persulfate (1:1, *v*/*v*), followed by incubation in the dark at 25 °C for 12–16 h. The solution was diluted with anhydrous ethanol to an absorbance of 0.700 ± 0.02 at 734 nm. For the assay, 0.5 mL of sample was mixed with 3.0 mL of ABTS solution, vortexed, and incubated at 25 °C for 30 min. Absorbance was measured at 734 nm, and the radical scavenging rate (%) was calculated as

$$Free\;radical\;scavenging\;rate\left(\%\right)=\frac{A_{5}-A_{6}}{A_{5}}\times 100$$

where A_5_ is the absorbance of the blank control, in which the sample is replaced by PBS, and A_6_ is the absorbance of the sample.

### 2.4. Safety Assessment

#### 2.4.1. Hemolysis Assay

Strains were streaked onto Columbia blood agar using sterile loops and incubated aerobically at 37 °C for 48 h (inverted). Hemolytic activity was assessed by examining hemolytic zones around colonies.

#### 2.4.2. Antibiotic Sensitivity Test

Antibiotic susceptibility was assessed using the Kirby–Bauer disk diffusion method against ten agents per European Food Safety Authority (EFSA) guidelines [14]: ampicillin (10 μg), streptomycin (10 μg), rifampicin (5 μg), erythromycin (15 μg), kanamycin (30 μg), penicillin G (10 μg), levofloxacin (5 μg), gentamicin (10 μg), chloramphenicol (30 μg), and tetracycline (10 μg). Bacterial lawns were prepared by spreading 0.1 mL of overnight cultures, adjusted to a turbidity of 0.5 McFarland standard, onto Mueller–Hinton agar (supplemented with 5% defibrinated sheep blood for fastidious strains). Three antibiotic discs were aseptically applied per plate. Following aerobic incubation at 37 °C for 48 h (inverted), inhibition zone diameters were measured to 0.1 mm precision using vernier calipers. The results are interpreted according to the species-specific breakpoints provided by EFSA [14]. *Staphylococcus aureus* ATCC 25923 was used as a quality control strain.

#### 2.4.3. Determination of Aminergic Activity

Biogenic amines were analyzed by high-performance liquid chromatography (HPLC) using a Waters Alliance HPLC system (Model e2695, Waters Technologies (Shanghai) Co., Ltd., Shanghai, China), following the method described by Chang et al. [15]. Briefly, 750 μL of chlordane derivatization reagent was added to 750 μL of sample extract at 4 °C in the dark (10 mg mL^−1^) and 150 μL of saturated Na_2_CO_3_ solution. The mixture was derivatized at 45 °C for 30 min, filtered (0.22 μm), and separated on a C18 reversed-phase column (5 μm, 4.6 mm × 250 mm) with UV detection.

### 2.5. Key Quality Attribute Assessment

#### 2.5.1. Determination of Diacetyl Content

The method for determining the content of diacetyl was modified according to Tian et al. [16]. Fermented milk samples (5.0 g) were homogenized with an equal volume of 16% (*w*/*v*) trichloroacetic acid. After 10 min of incubation at ambient temperature, mixtures were centrifuged at 4 °C for 5 min (8000× *g*). The supernatants were filtered through 0.45 μm membranes and stored at 4 °C pending analysis. For analysis, 5 mL of the treated sample was aliquoted into two parallel sets of test tubes. To the first set (Sample), 0.5 mL of 1% (*w*/*v*) p-phenylenediamine was added; the second set (Blank) received no additive. Following thorough vortex mixing, samples were incubated in the dark for 30 min. Reactions were terminated by adding 4.0 mol L^−1^ HCl: 2.0 mL to the sample tubes and 2.5 mL to the blank tubes. After homogenization, absorbance at 335 nm was recorded using a quartz cuvette, with the blank set as the reference solution.

#### 2.5.2. Determination of Proteolytic Capacity

Free amino acid content was quantified using o-phthalaldehyde (OPA) derivatization [8]. Fermented milk samples (5 mL) were treated with 10 mL of 0.75 mol L^−1^ trichloroacetic acid and 1 mL of distilled water. After homogenization, mixtures were incubated at room temperature for 10 min and then centrifuged at 4 °C for 10 min (6000× *g*) and filtered. Supernatant aliquots (50 μL) underwent reaction with 1 mL of OPA reagent for 2 min at room temperature. Absorbance was measured at 340 nm using a multifunction microplate reader (Thermo Scientific, Thermo Fisher Scientific, Finland) equipped with a quartz cuvette module. A standard curve was generated with leucine (0 to 0.5 mg mL^−1^), and sample-free amino acid concentrations were calculated based on the curve.

### 2.6. Physicochemical Property Analysis

#### 2.6.1. Texture Profile Analysis

Textural parameters, including firmness, cohesiveness, consistency, and index of viscosity, were determined by texture profile analysis (TPA) following the guidelines of ISO 11036:2020 and the instrumental TPA principle. The analysis was performed using a texture analyzer (Model TA.XT.plus, Stable Micro Systems Ltd., Godalming, UK) equipped with a Ø35 mm cylindrical probe (A-BE). Test speed was set at 1.00 mm s^−1^, with a deformation distance of 8.00 mm and a trigger force of 5.0 g. The TPA parameters were calculated automatically by the accompanying software (Exponent version 6.1.4.0).

#### 2.6.2. Texture and Water-Holding Capacity (WHC)

WHC was assessed by centrifuging 5× *g* of fermented milk in 15 mL tubes at 3000 rpm for 30 min. The supernatant was discarded, and the remaining pellet was weighed. WHC (%) was calculated as follows:

$$WHC\left(\%\right)=\frac{m_{3}-m_{1}}{m_{2}-m_{1}}\times 100$$

where m_1_ is the tube weight, m_2_ is the weight before centrifugation, and m_3_ is the weight after supernatant removal.

#### 2.6.3. Rheological Properties

Apparent viscosity was measured using a rheometer (Model Kinexus Pro+, Malvern Panalytical Ltd., Malvern, UK) with a PU40 parallel plate (gap 1.0 mm) at 25 °C. Shear viscosity was recorded over a shear rate range of 1–1000 s^−1^ to assess flow behavior.

### 2.7. Statistical Analysis

Data from at least three independent experiments are expressed as the mean ± standard error of the mean (SEM). Statistical comparisons were performed using IBM SPSS Statistics software (version 26.0, IBM Corp., Armonk, NY, USA). Specifically, after confirming normality and homogeneity of variance, a one-way analysis of variance (ANOVA) was conducted, followed by Duncan’s post hoc test for multiple comparisons. A *p*-value of <0.05 was considered statistically significant. Data visualization and further graphical analysis were conducted using GraphPad Prism (version 10.0.2, GraphPad Software, San Diego, CA, USA) and Origin (version 8.5, OriginLab Corporation, Northampton, MA, USA).

## 3. Results and Discussion

### 3.1. Key Fermentation Parameters of L. rhamnosus WH.FH-19 Fermented Milk

**Figure 1:**
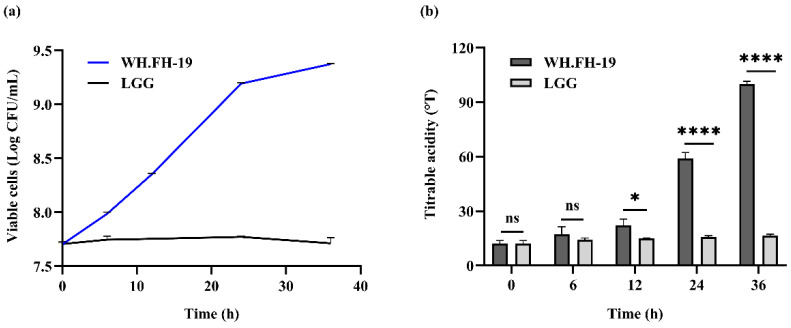
Determination of the number of viable bacteria and lactic acid content during fermentation: (**a**) viable cells; (**b**) titratable acidity. * *p <* 0.05, **** *p* < 0.0001 indicate significant differences, and ns indicates no significant differences. The error bars on the bar charts represented the standard deviations.

Viable lactic acid bacteria (LAB) counts in fermented milk must remain ≥10^6^ CFU mL^−1^ throughout shelf life, a critical quality benchmark for ensuring potential probiotic efficacy [17]. As shown in Figure 1a, *L. rhamnosus* WH.FH-19 colonies were enumerated in skim milk over a 36-h fermentation period to assess their growth kinetics. The viable count of *L. rhamnosus* WH.FH-19 increased progressively, exhibiting rapid growth within the first 24 h, followed by reduced proliferation thereafter. In contrast, the control strain LGG showed minimal fluctuation in viable counts within the milk matrix, *L. rhamnosus* WH.FH-19 exhibited significantly higher acidification than the LGG control from 12 h onward (*p* < 0.05), which indicated that there was accelerated accumulation of organic acids during later fermentation stages. The stability of the strain during refrigerated storage, confirming compliance with the aforementioned shelf-life criterion, is presented in Section 3.6.1.

The titratable acidity is a very important index for yogurt fermentation [18]. As shown in Figure 1b, the titratable acidity of fermented milk increased from 12.130 to 99.819 °T during 36 h of fermentation. *L. rhamnosus* WH.FH-19 exhibited rapid growth within 24 h, followed by slower proliferation, consistent with typical exponential-to-stationary phase transition dynamics. This strain demonstrated superior adaptation to milk components and conditions, indicating a potential for enhanced growth and beneficial metabolite yield. Conversely, LGG maintained stable viable counts in milk, suggesting constrained environmental adaptation. *L. rhamnosus* WH.FH-19’s enhanced milk matrix adaptation corresponded with greater acidification capacity relative to LGG. In the fermented milk, the lactose was hydrolyzed to glucose and galactose, and the glucose was converted to pyruvate by EMP, which was subsequently reduced to lactic acid [19]. Therefore, the higher TA value of *L. rhamnosus* WH.FH-19 may be due to its excellent lactose hydrolysis ability. In addition, *L. rhamnosus* WH.FH-19’s elevated acid output suggests potential for modifying fermented milk texture and sensory properties through targeted metabolite generation.

### 3.2. Adaptive Assessment of L. rhamnosus WH.FH-19 in the Gastrointestinal Tract

**Figure 2:**
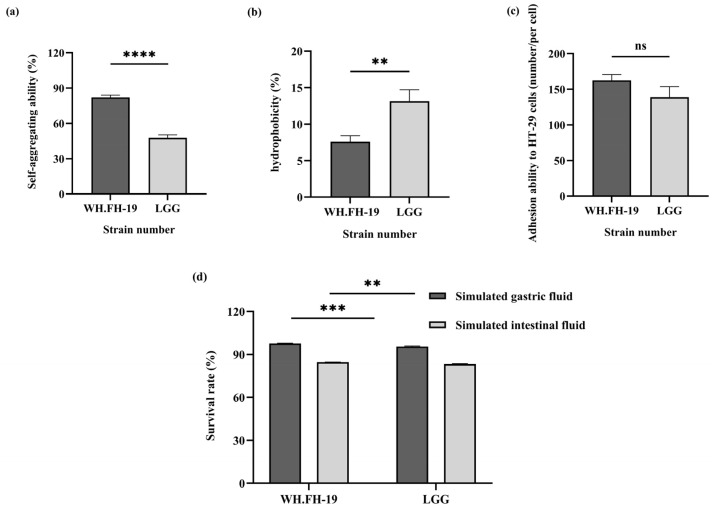
Evaluation of strain colonization potential and gastrointestinal tolerance: (**a**) auto-aggregation ability; (**b**) cell surface hydrophobicity; (**c**) adhesion to HT-29 cells; (**d**) survival in simulated intestinal fluid. **p < 0.01, *** *p* < 0.001, **** *p* < 0.0001 indicate significant differences; ns indicates no significant difference. Error bars represent standard deviations.

Enhanced auto-aggregation promotes stable microbial community establishment in host intestines while reducing exposure to adverse conditions [20]. Hydrophobicity serves as a critical probiotic selection criterion, mediating strain aggregation, biofilm formation, and initial host-cell adhesion [21]. As shown in Figure 2a,b, *L. rhamnosus* WH.FH-19 exhibited significantly higher auto-aggregation capacity (*p* < 0.05) yet lower cell surface hydrophobicity (*p* < 0.05) compared to the LGG control. This result indicated that *L. rhamnosus* WH.FH-19 demonstrated a distinct colonization mechanism balancing community stability with potentially altered host-interaction dynamics. This resilience supports the potential for intestinal colonization, a fundamental requirement for probiotic functionality.

Probiotic efficacy requires bacterial adhesion to intestinal mucus or epithelial cells for prolonged persistence, which directly governs colonization potential. Given the challenges of in vivo adhesion assessment in lactic acid bacteria, the HT-29 cell models provide a validated in vitro evaluation of probiotic traits [22]. As shown in Figure 2c, *L. rhamnosus* WH.FH-19 adhesion capability remained robust, with 162 adherent cells per field versus LGG’s 139 (*p* > 0.05). Although this difference did not reach statistical significance (*p* > 0.05), the robust adhesion performance suggests a potential for *L. rhamnosus* WH.FH-19 to competitively exclude pathogens by occupying mucosal binding sites. Therefore, the biological relevance of this trend should not be dismissed, as it indicates that *L. rhamnosus* WH.FH-19 possesses adhesion properties comparable to, and potentially even more effective than, the well-established probiotic strain LGG.

Metabolic activity maintenance during gastrointestinal transit is essential for probiotic strains to exert beneficial functions, including microbiota modulation [23] and intestinal immune enhancement [24]. As shown in Figure 2d, *L. rhamnosus* WH.FH-19 exhibited significantly higher tolerance to simulated gastric conditions (pH 3.0 for 3 h) compared to the reference strain LGG, with survival rates of 97.76% and 95.51%, respectively (*p* < 0.05). This survival rate exceeds the >90% acid tolerance threshold established for probiotic *L. rhamnosus* strains [25], confirming *L. rhamnosus* WH.FH-19’s robust gastric tolerance. Bile salt concentration and exposure duration critically govern bacterial tolerance in this harsh environment [26]. Following gastric incubation, both strains were exposed to simulated intestinal fluid (pH 8.0 with 0.3% bovine bile salts) for 8 h. Under these conditions, *L. rhamnosus* WH.FH-19 and LGG showed comparable survival rates of 84.55% and 83.20%, with no significant difference. It indicated that *L. rhamnosus* WH.FH-19 demonstrated robust intestinal fluid tolerance (>80% survival), indicating the capacity to withstand physiological fluctuations during gastrointestinal transit. These attributes position *L. rhamnosus* WH.FH-19 is a promising candidate where biofilm formation and mucosal adherence are critical probiotic determinants.

### 3.3. Cholesterol-Lowering and Antioxidant Capacity

**Figure 3:**
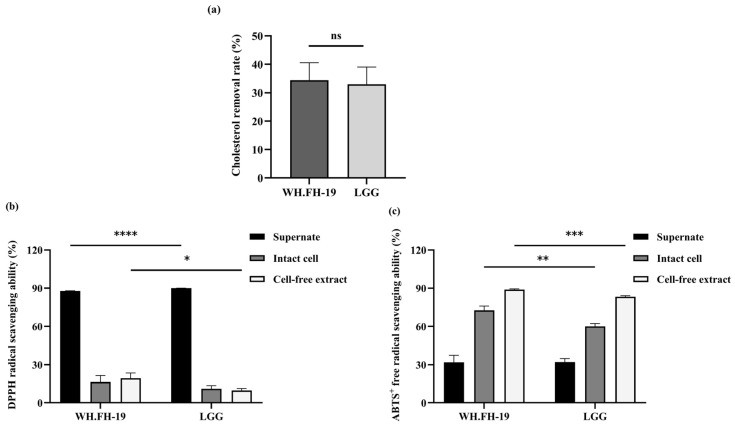
The cholesterol-lowering ability and the free radical scavenging ability of *L. rhamnosus* WH.FH-19: (**a**) cholesterol-lowering capacity; (**b**) DPPH radical scavenging ability; (**c**) ABTS+ free radical scavenging ability. * *p* < 0.05, ** *p* < 0.01, *** *p* < 0.001, **** *p* < 0.0001 indicate significant differences, and ns indicates no significant differences. The error bars on the bar charts represented the standard deviations.

Cholesterol-lowering activity represents a critically important phenotypic trait utilized as a key selection parameter for probiotic strains purported to possess health-promoting properties, particularly within the context of cardiovascular risk modulation [27]. This emphasis stems from the well-established pathophysiological link wherein elevated serum cholesterol concentrations, specifically elevated levels of low-density lipoprotein cholesterol (LDL-C), constitute a major modifiable risk factor driving the development and progression of atherosclerosis, thereby significantly increasing the incidence of adverse cardiovascular events [28]. Significant heterogeneity in cholesterol assimilation capacity was observed across the bacterial strains examined. Specifically, *L. rhamnosus* WH.FH-19 exhibited a capacity for cholesterol uptake that was quantitatively comparable to that measured for the reference LGG. This observed equivalence in cholesterol assimilation levels between *L. rhamnosus* WH.FH-19 and the LGG control was statistically non-significant (Figure 3a), as determined by appropriate comparative analysis (*p* > 0.05). This observed functional equivalence between *L. rhamnosus* WH.FH-19 and the established probiotic reference strain LGG in cholesterol assimilation, despite broader inter-strain heterogeneity, suggests *L. rhamnosus* WH.FH-19’s potential utility as a candidate for cholesterol-lowering applications, pending further mechanistic and in vivo validation.

Elevated DPPH/ABTS radical-scavenging capacity indicates that probiotic-fermented milk, or the bioactive compounds it generates, can effectively neutralize free radicals in vitro. DPPH radical scavenging capacity varied significantly among bacterial fractions (Figure 3b), with cell-free supernatant demonstrating superior activity versus intact cells and cell-free extracts. *L. rhamnosus* WH.FH-19 significantly outperformed LGG only in the cell-free extract (*p* < 0.05), with no differences in the other fractions. This hierarchy reflects bioactive metabolites secreted into the supernatant during bacterial growth, which exhibit radical quenching and oxidative stress mitigation properties [29]. These findings align with reported DPPH scavenging patterns across nine lactic acid bacteria strains [30]. ABTS^+^ scavenging assays revealed distinct fraction-dependent activity patterns (Figure 3c), with cell-free supernatant demonstrating reduced capacity compared to both intact cells and cell-free extracts. *L. rhamnosus* WH.FH-19 exhibited significantly greater ABTS^+^ scavenging in both cell-free extracts and intact cells versus LGG (*p* < 0.05), though supernatants showed no inter-strain difference (*p* > 0.05). This inverse hierarchy relative to DPPH radical quenching aligns with prior observations [31] and reflects fundamental radical properties: ABTS^+^ (hydrophilic) versus DPPH^+^ (hydrophobic) reactivity profiles [32]. Intact cell activity suggests surface-localized antioxidants, consistent with findings that removing surface proteins or polysaccharides diminishes radical scavenging in lactobacilli [33]. Collectively, *L. rhamnosus* WH.FH-19 demonstrates superior antioxidant capacity across multiple radical systems.

### 3.4. Safety Evaluation of L. rhamnosus WH.FH-19

**Figure 4:**
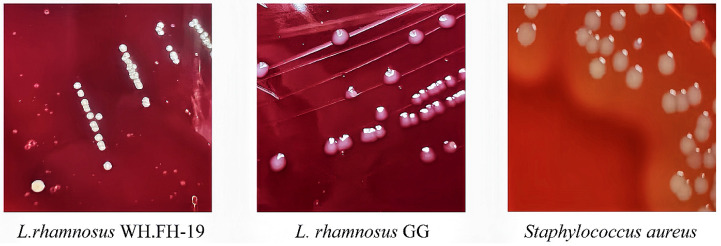
Hemolysis of *L. rhamnosus* WH.FH-19 and *L. rhamnosus* LGG.

Safety assessment revealed no detectable hemolytic activity for either *L. rhamnosus* WH.FH-19 or the reference LGG, in clear contrast to the pronounced hemolysis exhibited by the positive control *Staphylococcus aureus* (Figure 4). The strain displayed moderate susceptibility to the DNA gyrase inhibitor levofloxacin (Table 1). Comprehensive antibiotic susceptibility profiling demonstrated that *L. rhamnosus* WH.FH-19 exhibits sensitivity to inhibitors targeting essential bacterial processes: specifically, it was susceptible to multiple protein synthesis inhibitors (streptomycin, erythromycin, chloramphenicol, tetracycline) and cell wall synthesis inhibitors (ampicillin, penicillin G). Moderate susceptibility to levofloxacin was observed, while resistance to kanamycin and gentamicin was noted, consistent with intrinsic resistance patterns commonly reported in *Lactobacillus* spp. [22]. Critical analysis of biogenic amine production confirmed the absence (below the limit of detection) of histamine, spermidine, and spermine (Table 1). Measurable tyramine levels were precisely quantified at 4.853 mg L^−1^, a concentration significantly below the stringent 100 mg L^−1^ safety threshold established by the Food and Drug Administration (FDA). Trace phenylethylamine, putrescine, and cadaverine were present at biologically insignificant levels, confirming low decarboxylation activity relative to biogenic amine-producing food lactobacilli [34]. This favorable amine profile highlights the strain’s low decarboxylation activity and minimal risk of amine-related adverse effects. These results confirm the biosafety of *L. rhamnosus* WH.FH-19 and support its suitability as a probiotic candidate for food applications.

### 3.5. Technological Optimization

Diacetyl content serves as a key indicator for assessing dairy product quality [35]. This flavor compound, characterized by its distinctive buttery aroma, represents a key sensory component in fermented dairy products. Textural characteristics are widely recognized as important indicators of fermented milk quality. Among them, hardness refers to the maximum force required to compress the sample to a defined depth and is considered a key parameter in assessing product quality [36]. Cohesiveness reflects the intrinsic bonding strength between molecules within the sample structure. Therefore, we utilized diacetyl content and texture characteristics as dual metrics. We performed single-factor experiments to optimize the fermentation parameters for a mixed-strain culture of *L. rhamnosus* WH.FH-19 (LR), *L. bulgaricus* CICC 6047 (LB), and *S. thermophilus* CICC 6038 (ST).

**Figure 5:**
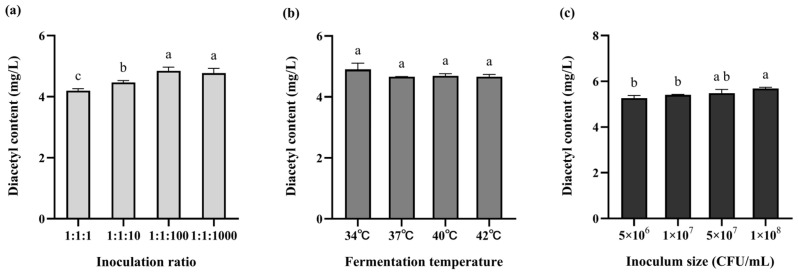
Diacetyl content of fermented milk under different fermentation conditions: (**a**) proportion of vaccination; (**b**) fermentation temperature; (**c**) amount of inoculation. The different lowercase letters in the figure indicate significant differences in the content of diacetyl (*p* < 0.05).

Diacetyl production in fermented milk increased with rising *L. rhamnosus* WH.FH-19 inoculation levels, showing a clear dose-dependent trend (Figure 5a). Starter culture ratios had a significant effect on diacetyl yield (*p* < 0.05), with both 1:1:100 and 1:1:1000 combinations outperforming other ratios. In texture analysis (Table 2), the starter ratio of 1:1:100 (LB:ST:LR) resulted in the highest hardness value. At this ratio, both cohesiveness and viscosity index were significantly higher than those observed at 1:1:1000 (*p* < 0.05). These findings indicate that 1:1:100 was the optimal inoculation ratio. The strong lactose utilization capacity of the base starter culture establishes it as the dominant strain, potentially inhibiting the growth of the probiotic adjunct *L. rhamnosus* WH.FH-19. To promote *L. rhamnosus* WH.FH-19 dominance and maximize its probiotic benefits, its inoculation proportion was intentionally increased beyond that of the base culture.

In contrast, fermentation temperature showed no significant impact on diacetyl synthesis (*p* > 0.05) (Figure 5b). Texture measurements (Table 3) revealed no significant differences among 37 °C, 40 °C, and 42 °C (*p* > 0.05), while samples fermented at 34 °C exhibited significantly lower texture values (*p* < 0.05). Additionally, coagulation time was noticeably prolonged at 34 °C, likely due to reduced growth and metabolic activity of the strains at lower temperatures. These results suggest that 40 °C is a more suitable fermentation temperature for *L. rhamnosus* WH.FH-19.

Inoculum concentration also had a substantial impact. The highest diacetyl production was observed at an LR concentration of 1 × 10^8^ CFU mL^−1^ (Figure 5c). Similar trends were observed in texture properties (Table 4), where increasing inoculation levels led to gradual increases in product hardness. No significant differences were found between 5 × 10^7^ CFU mL^−1^ and 1 × 10^8^ CFU mL^−1^ groups (*p* > 0.05), indicating that 1 × 10^8^ CFU mL^−1^ is the optimal inoculum level.

Based on these orthogonal optimization experiments, the optimal fermentation conditions were determined as follows: starter culture ratio LB:ST:LR = 1:1:100; fermentation temperature = 40 °C; inoculation concentrations = LB and ST at 1 × 10^6^ CFU mL^−1^, LR at 1 × 10^8^ CFU mL^−1^.

### 3.6. Fermentation Characteristics and Quality Evolution of L. rhamnosus WH.FH-19 Fermented Milk During the Fermentation and Storage Stages

#### 3.6.1. Acidification Dynamics and Post-Acidification Trend

**Figure 6:**
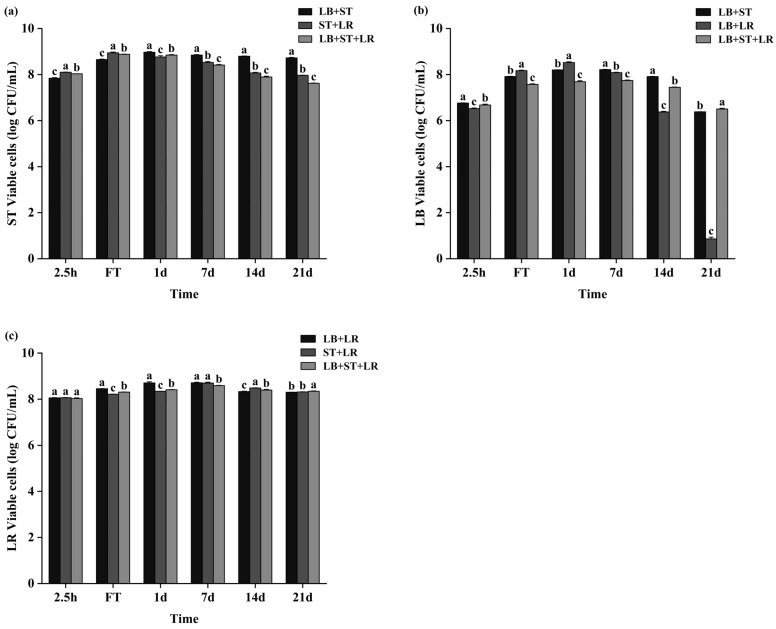
Viable bacterial counts of different strains during fermentation and subsequent storage. Panels show the changes in viable counts of (**a**) *S. thermophilus* CICC 6038 (ST), (**b**) *L. bulgaricus* CICC 6047 (LB), and (**c**) *L. rhamnosus* WH.FH-19 (LR) in fermented milk over the fermentation period and the following 21-day storage period at 4 °C. Within each panel, data points marked with different lowercase letters indicate statistically significant differences (*p* < 0.05) at the corresponding time points across groups.

The viable lactic acid bacteria (LAB) count is a critical quality metric for fermented milk. Consistent biphasic kinetics were observed during the 21-day storage across all fermentation groups, characterized by initial proliferation followed by a progressive decline (Figure 6), with post-storage minima quantified as ≥7.6 log CFU mL^−1^ for ST, ≥6.3 log CFU mL^−1^ for LB (except for the LB+LR co-culture group), and ≥8.3 log CFU mL^−1^ for LR, wherein the LB+LR co-culture uniquely demonstrated a marked divergence through substantially reduced LB viability after 21 days.

This process generates excessive sourness and sensory deterioration. Initial acidity measurements, uniformly approximating 15 °T across groups, underwent a steady decrease within the initial 2.5 h interval. The LB+ST+LR consortium consistently registered significantly higher acidity values relative to the LB+ST control formulation throughout this phase (*p* < 0.05) (Table 5), demonstrating LR’s capacity to augment collective metabolic flux. Subsequent monitoring documented a rapid acceleration in acidification, temporally coincident with the concurrent accumulation of organic acids, culminating in coagulation. Divergent acid production rates among the distinct formulations resulted in statistically significant differences in their respective fermentation endpoints, as enumerated in Table 6. Post-acidification results from the continued metabolic activity of lactic acid bacteria. Throughout the post-fermentation storage period, formulations supplemented with LR maintained significantly elevated acidity levels compared to LR-free control groups (*p* < 0.05) (Table 5). Notably, the LB+LR formulation exhibited pronounced progressive acidification during storage, reaching a terminal value of 124.14 °T by day 21. During storage, LB+LR exhibited significant progressive acidification. This phenomenon, attributed to continuous organic acid accumulation at refrigeration temperatures, poses product stability challenges [37]. Contrastingly, prior observations under analogous starter system conditions reported no exacerbation of post-acidification for the strongly acidogenic strain *Lactobacillus rhamnosus* PRA331 versus the weaker acidifier *Lactobacillus casei* PRA205 [38]. This also indicates that *L. rhamnosus* WH.FH-19 has an inhibitory effect on the post-acidification of fermented milk products.

#### 3.6.2. Content of Free Amino Acids

**Figure 7:**
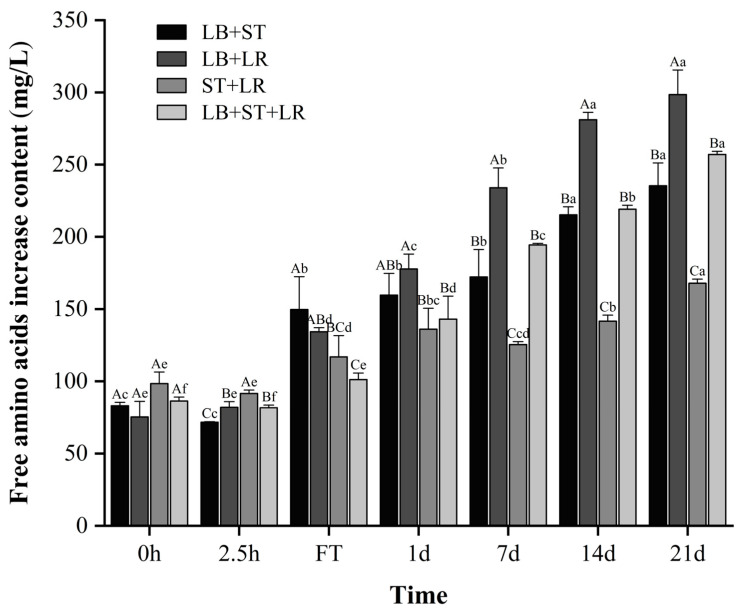
The content of free amino acids during fermentation and 21 days of storage. Values in the same column with different capital superscripts are significantly different (*p* < 0.05); values in the same row with different lowercase superscripts are significantly different (*p* < 0.05). LB, ST, and LR, respectively, represent *L. bulgaricus* CICC 6047, *S. thermophilus* CICC 6038, and *L. rhamnosus* WH.FH-19.

Microbial secretion of proteases and peptidases hydrolyzes milk proteins, liberating peptides and amino acids for cellular assimilation. These free amino acids serve dual functions: as indicators of proteolytic extent and as essential precursors for volatile flavor compound biosynthesis, enhancing both the nutritional value and sensory quality of the final product. Free amino acid (FAA) content progressively increased across all formulations during the 21-day fermentation and storage (Figure 7), with the LB+LR consortium exhibiting maximal accumulation in storage, consistent with reported proteolytic trends [39,40]. Temporal dynamics revealed significantly higher FAA in the LB+ST+LR group versus the LB+ST control at 2.5 h (*p* < 0.05). This indicates that LR possesses more efficient proteolytic activity, facilitating greater release of small peptides and amino acids, which may contribute to improved flavor, digestibility, and overall nutritional quality of the fermented product. This relationship was reversed at the fermentation terminus, where LB+ST surpassed LB+ST+LR in FAA content (*p* < 0.05), highlighting a critical kinetic trade-off between acidification and proteolysis. Under LB+ST+LR co-culture conditions, LR drives accelerated acidification kinetics, which likely suppresses proteolytic enzyme activity through pH-dependent denaturation within the suboptimal range for caseinolytic proteases. Conversely, the extended fermentation duration of the LB+ST consortium enables sustained protein hydrolysis despite slower initial kinetics, compensating for reduced early-stage proteolytic efficiency. This inversion correlates with LB+ST’s extended fermentation duration (Table 2), permitting greater proteolytic activity. Notably, no inter-group FAA differences emerged during storage (*p* > 0.05), potentially facilitated by its reduced acidity.

#### 3.6.3. Quality Evolution of Fermented Milk During Storage

Water-holding capacity (WHC) is a key indicator of the structural stability of protein gels in fermented milk [41]. Differences in WHC can also lead to distinct textural and sensory properties in dairy products [42]. As shown in Table 7, WHC varied among different fermentation combinations. At the end of fermentation and after 14 days of storage, the LB+ST+LR group showed significantly higher WHC than the LB+ST group (*p* < 0.05), suggesting that the addition of LR improved gel integrity and overall texture. The presence of LR may further enhance exopolysaccharides (EPS) production through potential synergistic interactions between the strains. These EPS can contribute to the formation of gel-like structures during fermentation, resulting in increased viscosity, improved mouthfeel, and enhanced water retention. During the 7–21-day storage period, the two groups containing LB and LR (LB+LR and LB+ST+LR) consistently exhibited higher WHC values. LB is commonly recognized as a strong producer of EPS, which acts as a natural bio-thickener that reduces syneresis and prevents moisture loss from food matrices [43]. This is one of the main reasons why the two groups of fermented milk with LB and LR have better water retention capacity, and this sustained WHC indicates superior textural stability in the final product.

In this part of the research, the structural properties of fermented milk were primarily evaluated based on hardness and cohesiveness. During storage, there was no significant difference (*p* > 0.05) in hardness and cohesiveness between the LB+LR and LB+ST+LR groups of fermented milk (Table 8). Consistent with previous findings reported by Ma et al. [14], both hardness and cohesiveness increased with prolonged storage, suggesting a gradual densification of the gel network and improved mouthfeel over time. During the storage period, no significant differences were observed between the LB+LR and LB+ST+LR groups in terms of hardness and cohesiveness (*p* > 0.05). This trend mirrors the results of the water-holding capacity analysis, indicating that the interaction between LB and LR may contribute more substantially to textural improvement than the presence of ST.

**Figure 8:**
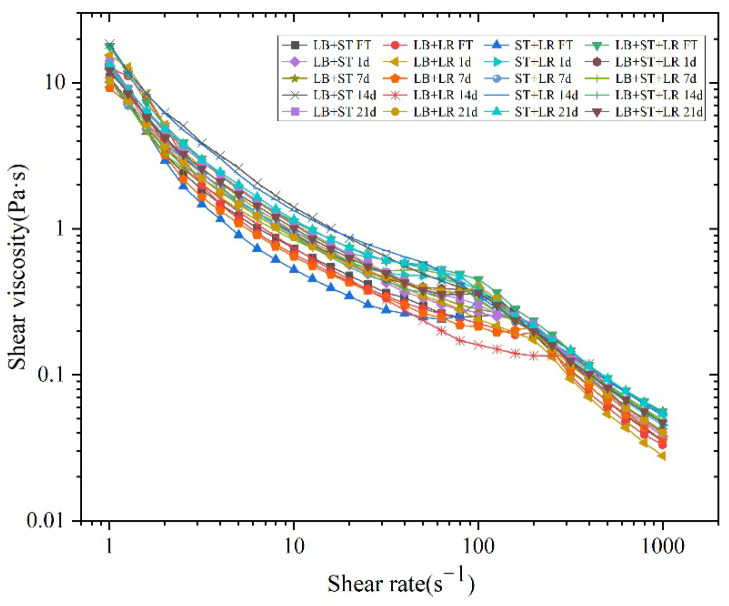
The change in shear viscosity with shear rate of fermented milk with different storage times.

Apparent viscosity is an important factor influencing the overall performance and consumer perception of fermented milk [44]. Rheological behavior is closely related to fluid viscosity, which in turn depends on shear rate. As shear rate increases, apparent viscosity tends to decrease, indicating shear-thinning behavior characteristic of pseudoplastic fluids [43]. As shown in Figure 8, all fermented milk samples exhibited shear-thinning behavior throughout the storage period. A slight increase in apparent viscosity was observed at a shear rate of 100 s^−1^, which may be attributed to the reorganization of the microstructure. This structural rearrangement likely led to a more compact network, resulting in increased resistance to flow.

## 4. Conclusions

This study qualifies *L. rhamnosus* WH.FH-19 is a future multifaceted probiotic starter with excellent technological and functional attributes for fermented milk foods. *L. rhamnosus* WH.FH-19 demonstrates superior potential as a multifunctional probiotic starter for fermented milk compared to LGG. *L. rhamnosus* WH.FH-19 exhibits accelerated acidification and enhanced growth in bovine milk, indicating superior dairy matrix adaptation and metabolic activity crucial for rapid coagulation and flavor development. *L. rhamnosus* WH.FH-19 possesses robust probiotic traits: good auto-aggregation, moderate hydrophobicity, strong adhesion to HT-29 cells, and high survival under simulated gastrointestinal conditions, supporting mucosal colonization and gut persistence. Functionally, *L. rhamnosus* WH.FH-19 matches LGG in cholesterol assimilation and demonstrates potent antioxidant activity (scavenging DPPH/ABTS radicals). Safety assessment confirms non-hemolytic activity, low biogenic amine production (tyramine below FDA limits), and clinically appropriate antibiotic sensitivity (intrinsic resistance only to aminoglycosides). Technologically, *L. rhamnosus* WH.FH-19 enhances diacetyl production, maintains high viability during cold storage, and improves water-holding capacity/texture (hardness, cohesiveness) in optimized co-cultures (e.g., LB:ST:LR = 1:1:100). While its in vitro and techno-functional profile is promising, in vivo validation of cholesterol-lowering efficacy, gut modulation, strain identity confirmation via sequencing, and industrial process optimization remain essential for commercial deployment.

## Figures and Tables

**Table 1:** Antibiotic sensitivity and biogenic amine production capacity of *L. rhamnosus* WH.FH-19.

	Antibiotic Sensitivity		Biogenic Amine (mg L^−1^)	
*L. rhamnosus* WH.FH-19	Ampicillin	S	Phenylethylamine	0.738 ± 0.189
	Streptomycin	S	Putrescine	0.336 ± 0.017
	Rifampicin	S	Cadaverine	0.959 ± 0.047
	Erythromycin	S	Histamine	ND
	Kanamycin	R	Tyramine	4.853 ± 0.647
	Penicillin G	S	Spermine	ND
	Levofloxacin	I	Spermidine	ND
	Gentamicin	R	Total Amines	6.886 ± 0.9
	Chloramphenicol	S		
	Tetracycline	S		

S: sensitive; I: moderately sensitive; R: resistant; ND: not detected.

**Table 2:** Texture determination of fermented milks with different inoculation ratios.

Inoculation Ratio	Firmness (g)	Consistency (g·s)	Cohesiveness (g)	Index of Viscosity (g·s)
1:1:1	104.52 ± 0.88 ^c^	654.82 ± 4.61 ^c^	−36.85 ± 1.35 ^a^	−212.90 ± 1.04 ^b^
1:1:10	114.53 ± 1.53 ^b^	720.53 ± 5.11 ^a^	−29.11 ± 0.99 ^b^	−224.30 ± 3.86 ^a^
1:1:100	129.53 ± 1.95 ^a^	702.10 ± 2.88 ^b^	−31.05 ± 1.10 ^b^	−154.52 ± 3.49 ^c^
1:1:1000	112.28 ± 2.55 ^b^	633.53 ± 8.74 ^d^	−19.28 ± 1.90 ^c^	−88.42 ± 1.27 ^d^

Values in the same column with different lowercase superscripts are significantly different (*p* < 0.05).

**Table 3:** Texture determination of fermented milks with different fermentation temperatures.

Temperature (°C)	Firmness (g)	Consistency (g·s)	Cohesiveness (g)	Index of Viscosity (g·s)
34	80.37 ± 13.19 ^b^	495.90 ± 67.34 ^b^	−23.47 ± 6.30 ^b^	−94.02 ± 36.63 ^b^
37	123.05 ± 7.69 ^a^	702.80 ± 31.32 ^a^	−38.6 ± 6.40 ^a^	−203.72 ± 41.04 ^a^
40	131.76 ± 17.89 ^a^	754.91 ± 50.87 ^a^	−37.74 ± 1.33 ^a^	−191.18 ± 8.92 ^a^
42	118.51 ± 22.16 ^a^	725.49 ± 108.61 ^a^	−35.62 ± 8.64 ^a^	−182.19 ± 42.13 ^a^

Values in the same column with different lowercase superscripts are significantly different (*p* < 0.05).

**Table 4:** Texture determination of fermented milks with different inoculum amounts.

Inoculum Size (CFU mL^−1^)	Firmness (g)	Consistency (g·s)	Cohesiveness (g)	Index of Viscosity (g·s)
5 × 10^6^	125.50 ± 17.19 ^b^	748.08 ± 46.50 ^b^	−37.41 ± 0.65 ^a^	−214.11 ± 15.24 ^ab^
1 × 10^7^	124.50 ± 12.90 ^b^	715.76 ± 55.07 ^b^	−35.5 ± 3.88 ^a^	−178.67 ± 12.49 ^b^
5 × 10^7^	160.21 ± 5.18 ^a^	875.15 ± 5.52 ^a^	−41.86 ± 7.97^a^	−231.84 ± 32.68 ^a^
1 × 10^8^	167.27 ± 1.69 ^a^	895.26 ± 20.53 ^a^	−36.24 ± 1.46^a^	−205.65 ± 18.18 ^ab^

Values in the same column with different lowercase superscripts are significantly different (*p* < 0.05).

**Table 5:** The acidity value of fermented milk changes during the fermentation and storage period.

Sample	Fermentation Time (°T)			Storage Time (°T)			
	0 h	2.5 h	FT	1 d	7 d	14 d	21 d
LB+ST	15.29 ± 0.17 ^Af^	25.39 ± 0.30 ^Be^	64.33 ± 0.14 ^Cd^	72.47 ± 0.75 ^Cc^	82.31 ± 1.63 ^Cb^	84.13 ± 2.66 ^Dab^	86.07 ± 3.02 ^Da^
LB+LR	15.56 ± 0.15 ^Ag^	20.43 ± 1.03 ^Cf^	79.88 ± 0.89 ^Ae^	85.85 ± 1.42 ^Ad^	89.17 ± 2.42 ^Bc^	118.40 ± 0.54 ^Ab^	124.14 ± 0.67 ^Aa^
ST+LR	15.71 ± 0.36 ^Ag^	24.20 ± 1.05 ^Bf^	69.72 ± 0.87 ^Be^	76.44 ± 0.85 ^Bd^	83.86 ± 0.68 ^Cc^	90.52 ± 0.47 ^Cb^	104.04 ± 1.73 ^Ca^
LB+ST+LR	15.28 ± 0.27 ^Ag^	28.17 ± 0.78 ^Af^	68.87 ± 1.13 ^Be^	78.56 ± 1.81 ^Bd^	93.54 ± 0.82 ^Ac^	103.12 ± 3.25 ^Bb^	109.58 ± 1.37 ^Ba^

Values in the same column with different capital superscripts are significantly different (*p* < 0.05); values in the same row with different lowercase superscripts are significantly different (*p* < 0.05).

**Table 6:** The time required for the fermentation of different groups of fermented milk to be completed.

Group	LB+ST	LB+LR	ST+LR	LB+ST+LR
Fermentation time (h)	4.5	5	4.25	4

**Table 7:** Determination of water retention capacity of fermented milk.

Sample	LB+ST	LB+LR	ST+LR	LB+ST+LR
FT	40.839 ± 1.750 ^Dbc^	43.007 ± 3.721 ^Cb^	38.712 ± 0.962 ^Bc^	63.217 ± 1.090 ^Ba^
1 d	42.634 ± 1.953 ^Dd^	50.593 ± 2.056 ^Bc^	60.093 ± 0.576 ^Ab^	66.451 ± 0.223 ^ABa^
7 d	47.366 ± 1.978 ^Cc^	68.694 ± 6.137 ^Aa^	58.434 ± 1.512 ^Ab^	66.468 ± 3.649 ^ABa^
14 d	60.932 ± 4.107 ^Bb^	69.896 ± 1.956 ^Aa^	59.985 ± 1.687 ^Ab^	66.479 ± 0.767 ^ABa^
21 d	72.098 ± 0.584 ^Aa^	72.563 ± 1.502 ^Aa^	58.975 ± 1.427 ^Ac^	67.982 ± 0.254 ^Ab^

Values in the same column with different capital superscripts are significantly different (*p* < 0.05); values in the same row with different lowercase superscripts are significantly different (*p* < 0.05).

**Table 8:** The texture of fermented milk changed after fermentation and during storage.

Fermented Milk		LB+ST	LB+LR	ST+LR	LB+ST+LR
Firmness (g)	FT	47.75 ± 1.30 ^Bd^	47.97 ± 3.50 ^Bd^	53.82 ± 3.42 ^Bd^	60.15 ± 4.10 ^Ad^
	1d	57.27 ± 1.55 ^Cc^	66.41 ± 3.82 ^ABc^	60.95 ± 4.66 ^BCc^	68.94 ± 2.30 ^Ac^
	7d	81.08 ± 0.72 ^Ab^	70.39 ± 4.33 ^Bc^	69.90 ± 3.52 ^Bb^	74.22 ± 3.95 ^Bbc^
	14d	83.08 ± 2.01 ^Bab^	79.57 ± 3.77 ^Bb^	91.25 ± 3.87 ^Aa^	77.51 ± 3.54 ^Bb^
	21d	86.30 ± 3.68 ^Ba^	88.19 ± 4.66 ^ABa^	94.22 ± 3.31 ^Aa^	87.78 ± 2.42 ^ABa^
Consistency (g·s)	FT	309.73 ± 3.47 ^Be^	293.42 ± 3.87 ^Cd^	307.44 ± 3.60 ^Be^	344.94 ± 3.84 ^Ae^
	1d	380.45 ± 3.15 ^Cd^	436.50 ± 4.17 ^Bc^	368.17 ± 4.83 ^Dd^	447.23 ± 4.73 ^Ad^
	7d	519.23 ± 4.61 ^Ab^	439.87 ± 3.86 ^Cc^	413.98 ± 3.70 ^Dc^	464.09 ± 3.27 ^Bc^
	14d	512.70 ± 3.38 ^Ac^	509.79 ± 3.29 ^Ab^	510.48 ± 4.69 ^Ab^	496.33 ± 3.74 ^Bb^
	21d	529.90 ± 1.96 ^Ba^	545.45 ± 4.77 ^Aa^	523.94 ± 1.69 ^Ca^	548.15 ± 2.39 ^Aa^
Cohesiveness (g)	FT	−12.18 ± 1.03 ^ABc^	−8.10 ± 2.33 ^Cd^	−9.77 ± 0.43 ^BCd^	−13.69 ± 1.89 ^Ad^
	1d	−18.70 ± 2.59 ^Bb^	−23.69 ± 2.60 ^Ac^	−14.75 ± 2.55 ^Bc^	−24.06 ± 1.76 ^Ac^
	7d	−32.62 ± 3.13 ^Aa^	−31.43 ± 2.35 ^Ab^	−20.70 ± 3.20 ^Bb^	−30.78 ± 2.41 ^Ab^
	14d	−30.20 ± 2.66 ^Ba^	−37.99 ± 3.26 ^Aa^	−25.28 ± 2.57 ^Cab^	−30.32 ± 0.97 ^Bb^
	21d	−31.92 ± 2.84 ^BCa^	−39.06 ± 3.66 ^Aa^	−26.81 ± 2.99 ^Ca^	−34.28 ± 1.33 ^ABa^

Values in the same column with different capital superscripts are significantly different (*p* < 0.05); values in the same row with different lowercase superscripts are significantly different (*p* < 0.05).

## Data Availability

The original contributions presented in the study are included in the article. Further inquiries can be directed to the corresponding author.
